# Supplementary figures and images for: Multi-Season Genome-Wide Association Study Reveals Loci and Candidate Genes for Fruit Quality and Maturity Traits in Peach
Source: Plants (Basel). 2026 Jan 7;15(2):189. doi: 10.3390/plants15020189 (PMC12844748; doi:10.3390/plants15020189)

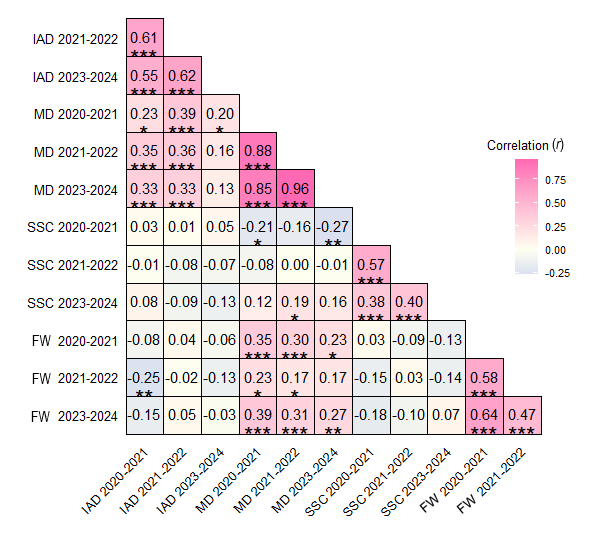

Supplement: Supplementary file 1 [file plants-15-00189-s001.zip › plants-3989010-supplementary-RESUBMIT.tif]
